# A simple method for bone age assessment: the capitohamate planimetry

**DOI:** 10.1007/s00330-017-5255-4

**Published:** 2018-01-30

**Authors:** Jung-Ah Choi, Young Chul Kim, Seon Jeong Min, Eun Kyung Khil

**Affiliations:** Department of Radiology, Hallym University Dongtan Sacred Heart Hospital, 7 Keunjaebong-gil, Hwaseong, 18450 Gyeonggi-do Republic of Korea

**Keywords:** Hand, Radiography, Child, Bone, Growth

## Abstract

**Objectives:**

To determine if the capitohamate (CH) planimetry could be a reliable indicator of bone age, and to compare it with Greulich-Pyle (GP) method.

**Methods:**

This retrospective study included 391 children (age, 1–180 months). Two reviewers manually measured the areas of the capitate and hamate on plain radiographs. CH planimetry was defined as the measurement of the sum of areas of the capitate and hamate. Two reviewers independently applied the CH planimetry and GP methods in 109 children whose heights were at the 50th percentile of the growth chart.

**Results:**

There was a strong positive correlation between chronological age and CH planimetry measurement (right, r = 0.9702; left, r = 0.9709). There was no significant difference in accuracy between CH planimetry (84.39–84.46 %) and the GP method (85.15–87.66 %) (p ≥ 0.0867). The interobserver reproducibility of CH planimetry (precision, 4.42 %; 95 % limits of agreement [LOA], −10.5 to 13.4 months) was greater than that of the GP method (precision, 8.45 %; LOA, −29.5 to 21.1 months).

**Conclusions:**

CH planimetry may be a reliable method for bone age assessment.

**Key Points:**

*• Bone age assessment is important in the work-up of paediatric endocrine disorders.*

*• Radiography of the left hand is widely used to estimate bone age.*

*• Capitatohamate planimetry is a reliable and reproducible method for assessing bone age.*

## Introduction

Bone age assessment (BAA) is crucial in the evaluation of endocrine disorders and in the prediction of adult height when hormone therapy is the treatment [1].

Radiographic examination is an easy and cost-effective method for the assessment of bone age. BAAs are performed using various body parts such as the hand, elbow, knee, cervical vertebra or pelvis [2]. Hand radiography is certainly the most widely used examination [3].

Several methods have been proposed, including Greulich-Pyle (GP) atlas, Tanner-White (TW) score and Gilsanz-Ratibin (GR) atlas, which are qualitative [4]. The TW method is based on the level of maturity for 20 regions of interest (ROIs), including the epiphysis, metaphysis and diaphysis in the radius, ulna, first, third and fifth fingers and the carpal bones, so it is complex and time-consuming. The GR method is another technique that uses the new digital atlas that includes reference images of the left hand taken at 6-month intervals from birth to age 6 years, and at 1-year intervals from age 7 to 17 years. However, this subjective method relies on matching of the hand radiograph of the subject with the GR atlas. In the GP method, bone age is determined by matching the left-hand radiograph of the subject with reference radiographs from the atlas. This explains why the GP method is generally easier and quicker to use, so it is more often selected over other methods [5–8]. One disadvantage of the GP method is the characteristics inherent in subjective analysis, and the interobserver reproducibility of BAA with the GP method has been controversial [5, 7].

A quantitative evaluation method with objective parameters is needed. BAA by using the capitate and hamate, which are the first bones to develop embryologically, has been recommended by a previous study but not performed as of yet [9]. In addition, validation of BAA across different ethnicities would be necessary for a more reliable method [10]. Therefore, the aim of this study was to determine if the area of the capitate and hamate bones measured on hand radiography could be a reliable indicator of bone age accuracy, precision, and reproducibility between the planimetry and GP methods.

## Materials and methods

### Populations

In this study, all data were collected from the electronic medical records of patients. From January 2014 to December 2015, 423 patients (age 1–180 months) presented to the emergency department. The study population was selected according to the following inclusion criteria: PA radiography of both hands, age younger than 15 years and belonging to a single ethnic group. The following patients were excluded: those with either only right-hand (n=42, 30 boys and 12 girls) or only left-hand (n=33, 24 boys and nine girls) PA radiograph, one with acute leukaemia (one boy) one with idiopathic hypertrophic pyloric stenosis (one boy) and those with other ethnicities (one Chinese, one Japanese and one American) [1, 10]. Finally, a total of 391 consecutive patients (242 boys and 149 girls) were enrolled in this study.

In total, we analysed 782 hands of 391 patients (242 boys and 149 girls). The chronological age, sex and ethnicity of the patients were recorded. The mean patient age was 5 years and 8.52 months (age range 1–180 months). The patients complained of contusion (n=222), laceration (n=175) and/or fracture (finger, 40; humerus, 54). The absence or presence of capitate and/or hamate bones of the hand was registered in all patients.

According to the growth chart published by the Korea Centers for Disease Control and Prevention (CDC) in 2007, among patients aged 1 month to 3 years, those whose height was between the 50th percentile of a child who is 1 month younger and the 50th percentile of a child who is 1 month older were selected [11]. Among patients aged 3–14 years, those whose height was between the 50th percentile of a child who is 6 months younger and the 50th percentile of a child who is 6 months older were selected [12]. A total of 109 children (mean age 113.07 months; standard deviation 36.07) were included in the 50th percentile group.

### Equipment and imaging protocol

Hand radiography was performed using Innovion-SH (DK Medical Systems, Seoul, Korea). The patients were seated alongside or facing a table, and examined in the PA position. The protocols used in this study for taking the PA radiographs of both hands are described in Table 1. Both hands were in neutral position with no flexion, extension or deviation, and were placed palm down on the cassette with the fingers extended [13].

**Table 1:** Imaging protocol of both hands posterior-anterior (PA) x-ray

Projection	Posterior-anterior bilateral projection
Detector size	24×30 cm
Orientation	Landscape
Exposure	50–60Kvp/2–5mAs
Grid	No
SID	100 cm
Central ray	Between the two hands at the level of the metacarpophalangeal joints
Collimation	To include one-third of the distal radius and ulna to include soft tissues

*SID* source to image-receptor distance

**Fig. 1:**
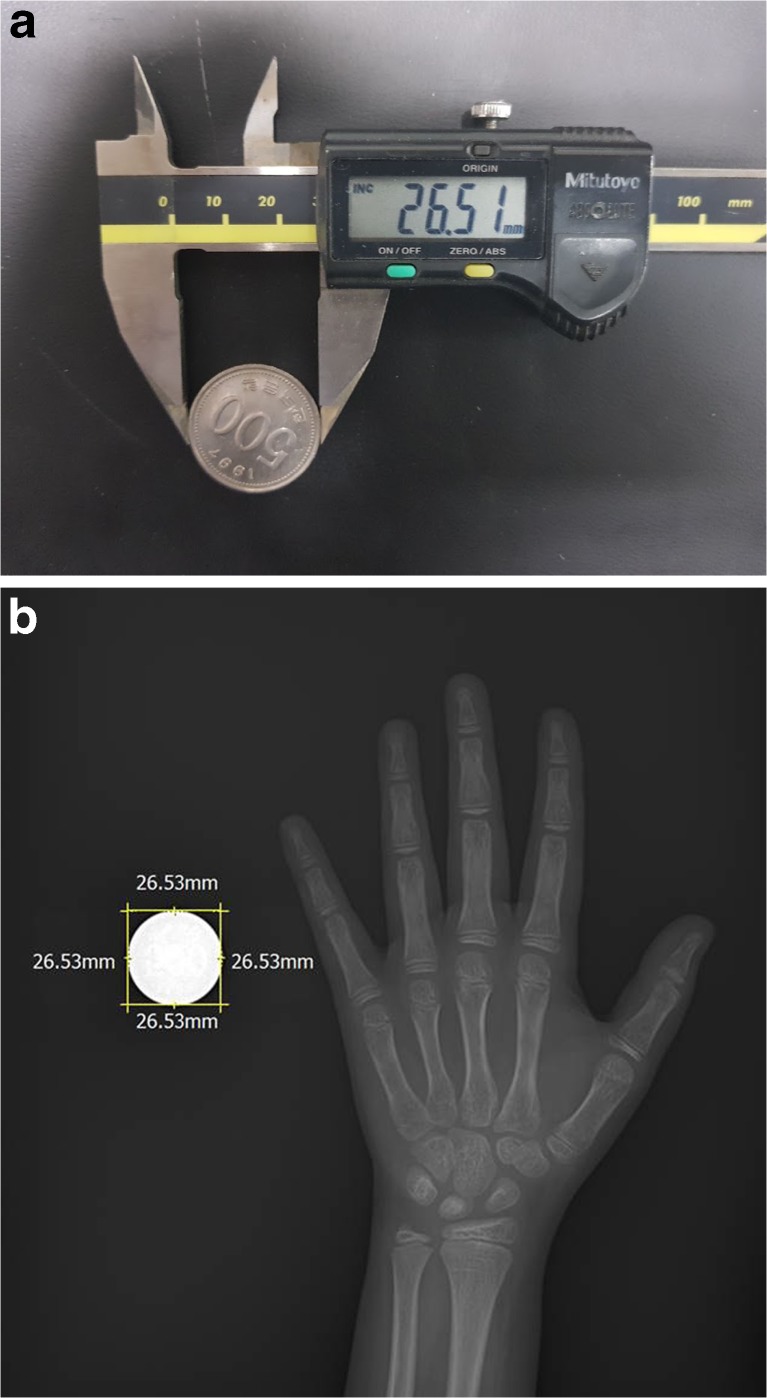
(**a**) The diameter of a coin measured using a digital micrometre was 26.51 mm. (**b**) The diameter of the coin on a digital radiograph was measured as 26.53 mm on a Picture Archiving and Communication System (PACS)

To assess reliability of measurements, calibration was performed using a coin (real value of the coin diameter, 26.51 mm; measured value of the coin diameter, 26.53 mm; relative error, 0.08 %). In this process, the source to image-receptor distance (SID) was set to 100 cm (Table 1). To prevent projection errors, the x-ray source was directed perpendicular to the cassette (Fig. 1).

### Image analysis

Four radiologists (three with 21, 18 and 12 years experience in radiology, respectively, and one first-year resident), blinded to the clinical data, analysed the images on a Picture Archiving and Communication System (PACS; G3, Infinitt Healthcare, Seoul, Korea).

**Fig 2:**
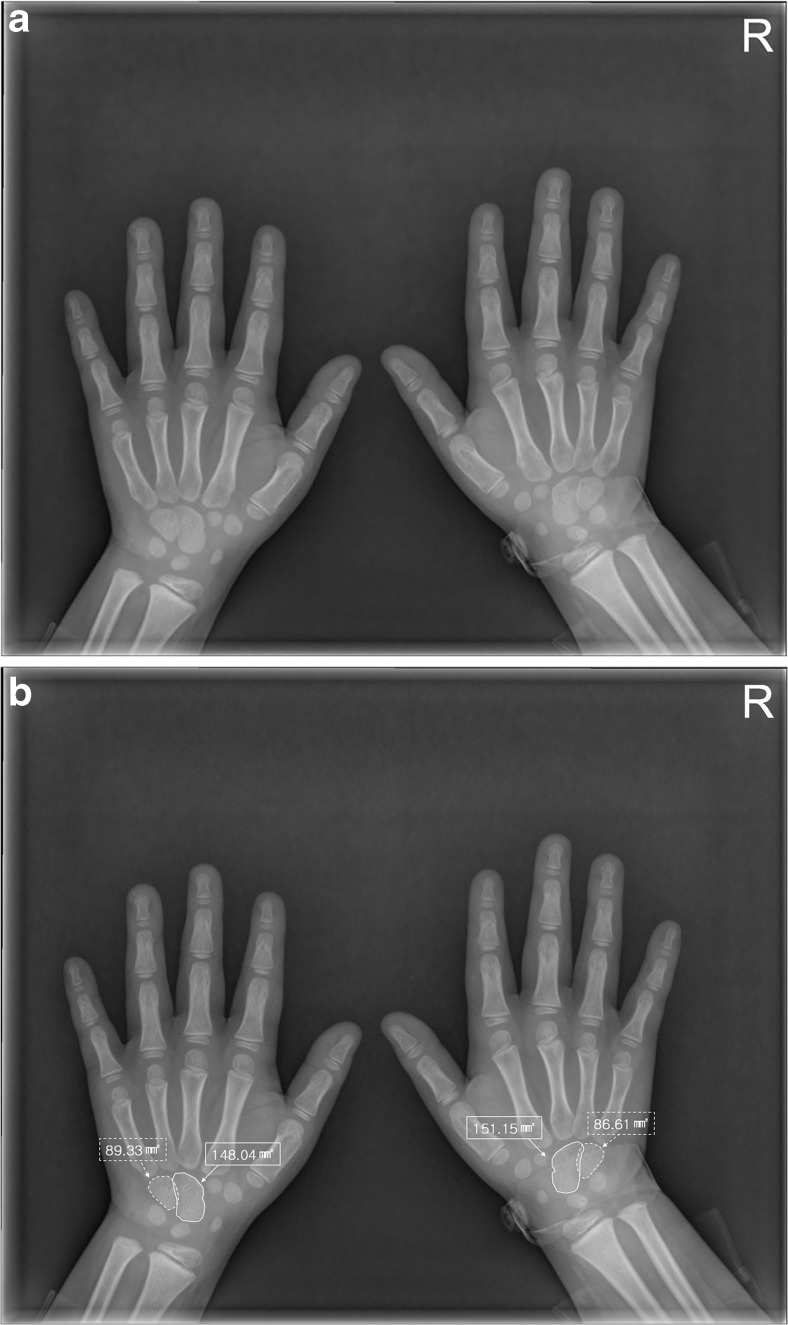
75-month (6 years and 3 months)-old boy. (**a and b**) The areas of the bilateral capitate bones (solid line) and bilateral hamate bones (dotted line) were measured by drawing two separate regions of interest in the posterior-anterior (PA) radiograph of both hands

For planimetric analysis, the first and second radiologists (with 18 and 12 years experience in radiology, respectively) independently measured the area of the capitate and the hamate on PACS, respectively. ROIs were drawn manually on plain radiographs to encompass the entire capitate and hamate separately. Area measurements of the capitate and hamate were performed for both right and left hands (Fig. 2).

When the ROI values were > 10 % different between the two reviewers, another series of measurements were performed by the same two reviewers to reach a consensus. Differences < 10 % were considered negligible, and the average was reported [7].

The CH planimetry measurement was calculated by summing the area of the capitate and that of the hamate as follows: CH planimetry = area of capitate + area of hamate. The values of CH planimetry were obtained from both hands.

For the analysis of accuracy and reproducibility for BAA, the third and fourth reviewers (with 21 years experience in radiology and the first-year resident, respectively) measured the area of the capitate and hamate of children who were included in the 50th percentile group. Each reviewer independently assessed the bone age of children using the CH planimetry method. On the basis of the GP method, the reviewers estimated the bone age of the 50th percentile group at 2 weeks after the planimetry assessment.

### Statistical analysis

All statistical analyses were conducted using SPSS (version 20; IBM Corporation). Linear regression analysis was used to analyse the relationship between the planimetry measurement of each hand and the chronological age of each sex. Correlation coefficients of ≤ 0.3 were considered to indicate a weak relationship; 0.30–0.70, moderate relationship; and > 0.70, strong relationship [14]. A z-test was used to compare two correlation coefficients.

Differences in the planimetry measurements between the right and left CH and in the accuracy and precision between two reviewers were analysed by using a paired t-test. A *p*-value of < 0.05 was considered statistically significant.

To compare the two methods, Deming regression analysis and accuracy determination were performed. Accuracy was calculated using the difference percentage between the estimated bone age and the chronological bone age of the 50th percentile group.

To assess the reproducibility of the measurements between the third and fourth reviewers, precision, Bland-Altman analysis and Lin’s concordance correlation coefficient (ρ_c_) were used. Precision was defined as the percentage difference in the estimated bone ages between two reviewers. The strength of ρ_c_ was categorized as follows: ρ_c_ < 0.90, poor agreement; 0.9 ≤ ρ_c_ ≤ 0.95, moderate; 0.95 < ρ_c_ ≤ 0.99, substantial; ρ_c_ > 0.99, almost perfect agreement.

## Results

The age and sex distribution of the Korean subjects are listed in Table 2. There were 242 boys (mean age 75.33 months; age range 1–180 months) and 149 girls (mean age 57.46 months; age range 1–178 months).

**Table 2:** Age and sex distribution of the individuals

Age (months)/Sex	Boys (n=242)	Girls (n=149)
1–11	13	9
12–23	37	31
24–35	33	17
36–47	21	26
48–59	16	13
60–71	14	6
72–83	17	12
84–95	6	5
96–107	13	5
108–119	10	7
120–131	4	3
132–143	18	4
144–155	14	3
156–167	9	5
168–179	16	3
180	1	0

Data represent number of patients

**Fig. 3:**
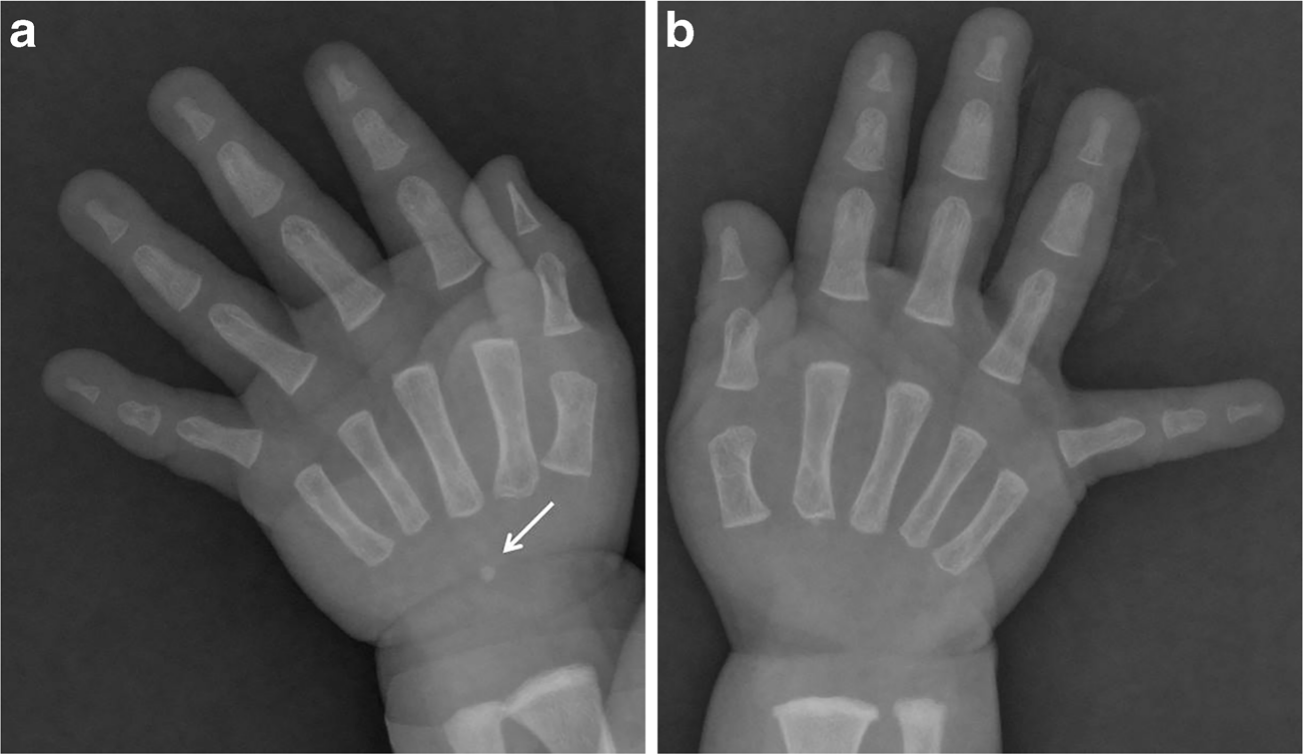
One-month-old boy. (**a and b**) Left capitate bone (arrow) is demonstrated but right capitate bone and bilateral hamate bones are not demonstrated on the posterior-anterior (PA) radiograph of both hands

For 241 boys (99.59 %) and 149 girls (100 %), irrespective of age, left and right capitate and hamate bones were demonstrated on the radiographs of both hands. The right capitate and bilateral hamates were not demonstrated on the radiograph in only one boy (0.41 %) aged 1 month (Fig. 3).

There were no significant differences in areas between the left and right capitate and hamate of both sexes (Table 3). Additionally, there was also no significant difference in the sum of the area of the capitate and the area of the hamate between right and left hands.

**Table 3:** Differences in the values of planimetry of boys and girls

Gender	Location	Right (95 % CI)	Left (95 % CI)	*p*-value*
Boy (n=242)	Capitate	130.52 (117.22–142.64)	129.94 (117.23-142.64)	0.3049
	Hamate	87.52 (78.48–96.57)	87.00 (78.07-95.93)	0.1691
	Capitohamate	218.04 (196.32–239.76)	216.94 (195.38-238.50)	0.1333
Girl (n=149)	Capitate	95.70 (84.00–107.41)	94.70 (83.17-106.22)	0.3611
	Hamate	64.69 (56.66–72.71)	64.16 (56.23-72.10)	0.2545
	Capitohamate	160.39 (140.75–180.02)	158.86 (139.53-178.20)	0.1779

*CI* confidence interval

*A paired t-test was used to assess the difference of the means

**Fig. 4:**
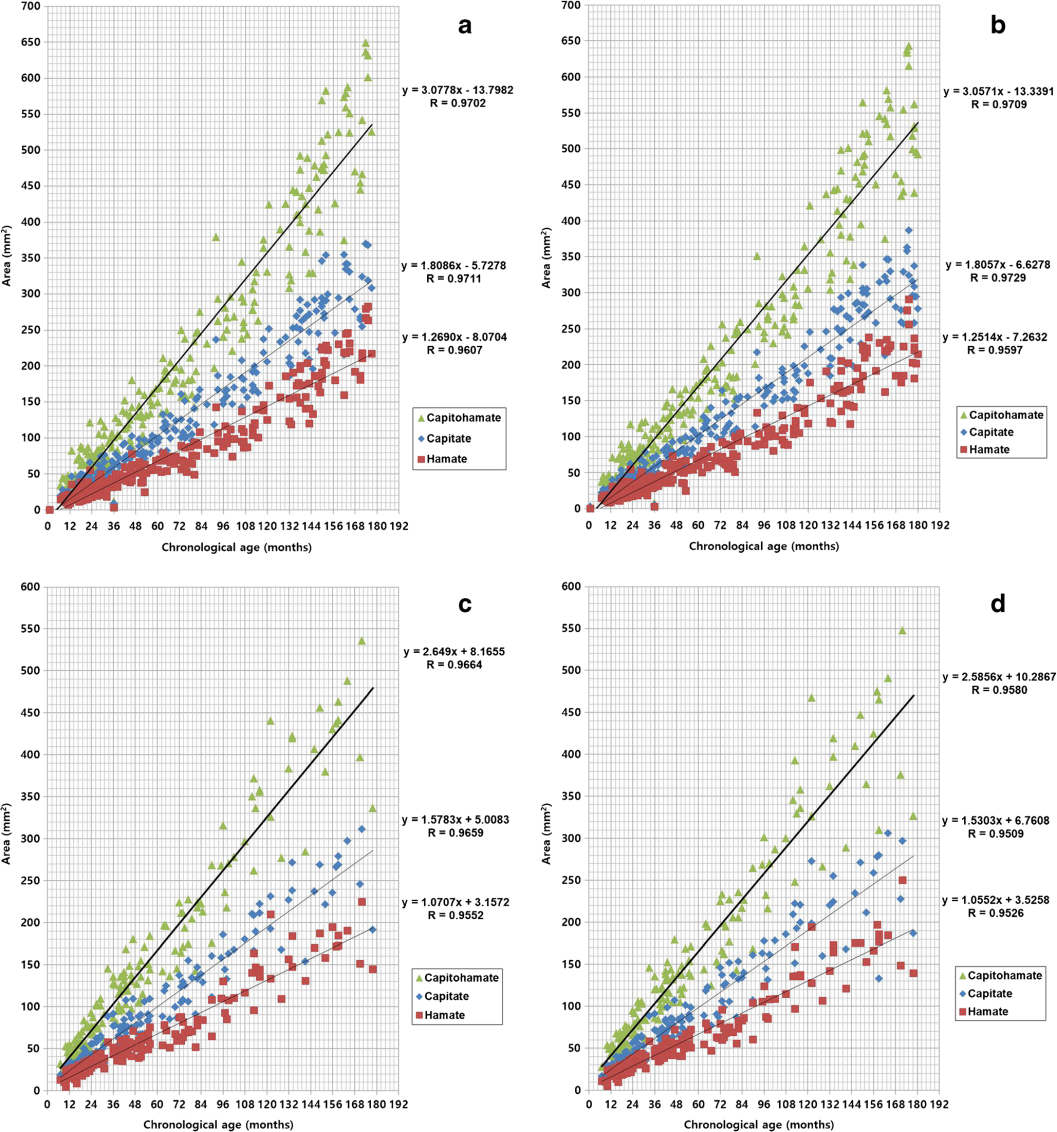
High positive relationships between chronological age and planimetries of capitates, hamates and CHs of the right (**a**) and left (**b**) hands of boys and right (**c**) and left (**d**) hands of girls. *CH* capitohamate

The planimetry curves of the capitate and hamate and the sum of the CH areas demonstrated increased slopes in both sexes (Fig. 4). There was a strong positive correlation between chronological age and the CH planimetry measurement (Table 4). Moreover, strong correlations were found between chronological age and the capitate area of both sexes, as well as between the chronological age and hamate area (Table 4).

**Table 4:** Differences in the correlation coefficients between planimetry and chronological age of boys and girls

Gender	Location	Right (95 % CI)	Left (95 % CI)	z-statistic
Boy	Capitate	0.9711 (0.9629–0.9775)	0.9729 (0.9652–0.9789)	0.9729
	Hamate	0.9607 (0.9496–0.9693)	0.9597 (0.9484–0.9686)	0.8886
	Capitohamate	0.9702 (0.9618–0.9768)	0.9709 (0.9627–0.9774)	0.8951
Girl	Capitate	0.9659 (0.9531–0.9752)	0.9509 (0.9327–0.9643)	0.1118
	Hamate	0.9552 (0.9386–0.9674)	0.9526 (0.9351–0.9655)	0.8052
	Capitohamate	0.9664 (0.9538–0.9756)	0.9580 (0.9424–0.9695)	0.3313

*CI* confidence interval

In the linear equation y = a + bx, y stands for capitate, hamate and CH area; x stands for age in months; and constants (a and b) were calculated. The equations of planimetry for the left hands of boys and girls are as follows:

$$ {\displaystyle \begin{array}{l}\mathrm{CH}\kern0.5em \mathrm{area}\kern0.5em \left(\mathrm{boy}\right)=3.0571\kern0.5em \mathrm{age}\kern0.5em \left(\mathrm{months}\right)-13.3391\\ {}\mathrm{CH}\kern0.5em \mathrm{area}\kern0.5em \left(\mathrm{girl}\right)=2.5856\kern0.5em \mathrm{age}\kern0.5em \left(\mathrm{months}\right)-10.2867\\ {}\mathrm{Capitate}\kern0.5em \mathrm{area}\kern0.5em \left(\mathrm{boy}\right)=1.8057\kern0.5em \mathrm{age}\kern0.5em \left(\mathrm{months}\right)-6.6278\\ {}\mathrm{Capitate}\kern0.5em \mathrm{area}\kern0.5em \left(\mathrm{girl}\right)=1.5303\kern0.5em \mathrm{age}\kern0.5em \left(\mathrm{months}\right)-6.7608\\ {}\mathrm{Hamate}\kern0.5em \mathrm{area}\kern0.5em \left(\mathrm{boy}\right)=1.2514\kern0.5em \mathrm{age}\kern0.5em \left(\mathrm{months}\right)-7.2632\kern0.5em \\ {}\mathrm{Hamate}\kern0.5em \mathrm{area}\kern0.5em \left(\mathrm{girl}\right)=1.0552\kern0.5em \mathrm{age}\kern0.5em \left(\mathrm{months}\right)-3.5258\end{array}} $$

By using the CH planimetry and GP methods, the third and fourth reviewers estimated the bone age of 109 patients who were classified into the 50th percentile group. None of the mean areas of the capitate, hamate and sum of the capitate and hamate were significantly different between the two reviewers. The mean value of chronological age of 109 children (mean age 113.07 months; standard deviation 36.07) was not significantly different from that of the estimated bone age measured by both CH planimetry and GP methods (p ≥ 0.1378). There was no statistically significant difference in the mean value of bone age between the two reviewers using CH planimetry. However, the mean values of bone age were significantly different between the two reviewers assessing the bone age according to the GP atlas (Table 5). The mean value of bone age estimated using the GP method was lower than that measured using CH planimetry (*p* < 0.0001).

**Table 5:** Descriptive statistics of the area and bone age estimated by two reviewers in the 50th percentile group

	Reviewer 3	Reviewer 4	*p*-value*
Area (mm^2^)			
Capitate	185.94 ± 65.42	184.11 ± 67.97	0.1128
Hamate	121.45 ± 44.63	120.25 ± 46.23	0.2487
Sum	307.39 ± 108.92	304.36 ± 112.82	0.0729
BAA (months)			
CH planimetry	118.58 ± 39.52	117.43 ± 40.96	0.0687
GP method	105.49 ± 39.00	109.49 ± 39.38	0.0003

Data are means ± standard deviation

*A paired t test was used to assess the difference of the means

*BAA* bone age assessment, *CH* capitohamate, *GP* Greulich-Pyle atlas method

**Fig. 5:**
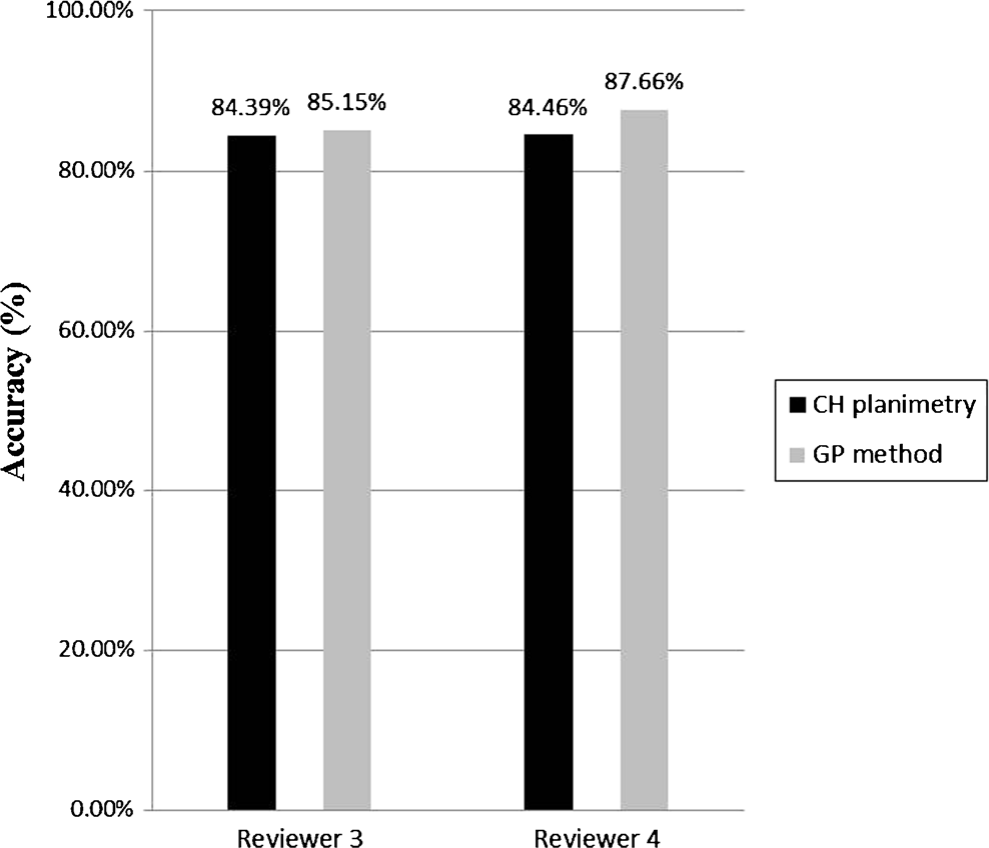
Graph showing accuracy of CH planimetry and GP method in assessing the bone age of 50th percentile group between two reviewers. A paired t-test demonstrated no statistically significant differences between two methods and between two reviewers (*p*-value ≥ 0.0867). Accuracy was defined as the difference percentage between the estimated bone age and the chronological bone age of 50 percentile group. *CH* capitohamate, *GP* Greulich-Pyle

**Fig. 6:**
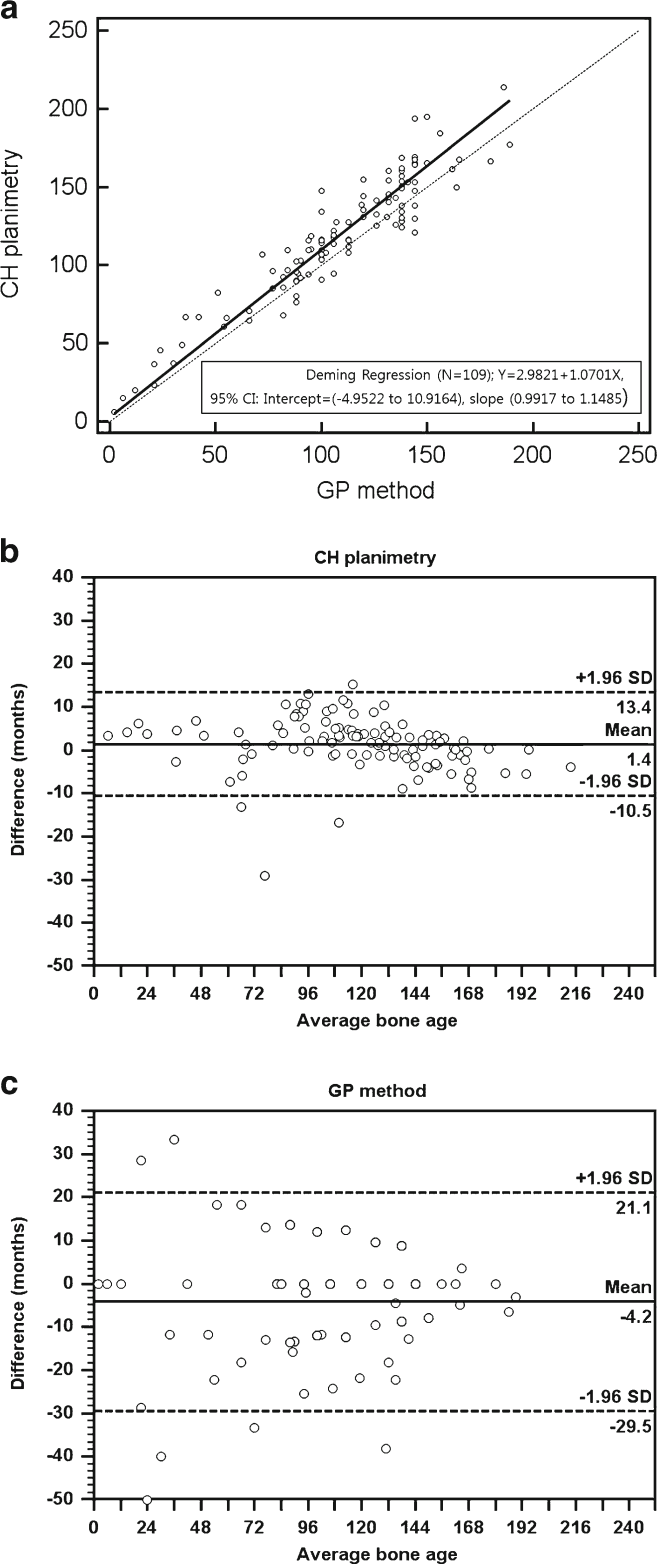
(**a**) Deming regression analysis for comparison of CH planimetry and GP atlas method in assessing bone age. (**b and c**) Bland-Altman plots of two reviewers show the interobserver reproducibility for CH planimetry and GB method for bone age assessment. The 95 % limits of agreement were narrower in CH planimetry than in GP method. *CH* capitohamate, *GP* Greulich-Pyle

When assessing bone age based on the CH planimetry and GP methods, there was no significant difference in the accuracy between the third (CH planimetry, 84.39 % ± 22.33 %; GP method, 85.15 % ± 13.78 %) and fourth (CH planimetry, 84.46 % ± 21.29 %; GP method, 87.66 % ± 12.34 %) reviewers (*p* ≥ 0.0867) (Fig. 5). Deming regression analysis showed no statistically significant difference in the results between the CH planimetry and GP methods, as the standard errors can be determined from the 95 % confidence intervals (CIs) of intercept (standard error, 4.0028; 95 % CI −4.9522–10.9164) and slope (standard error, 0.03955; 95 % CI, 0.9917–1.1485) including the values 0 and 1, respectively (Fig. 6).

We analysed the interobserver variability of the CH planimetry and GP methods. The values of ρ_c_ were substantial for both methods for assessing bone age (CH planimetry, 0.9862; GP method, 0.9553) between the two reviewers. However, the precision of CH planimetry (4.42 % ± 3.87 %) was significantly different from that of the GP method (8.45 % ± 8.52 %) (*p* < 0.001). The Bland-Altman analysis demonstrated that CH planimetry had narrower 95 % limits of agreement (−10.5 to 13.4 months) than the GP method (−29.5 to 21.1 months) (Fig. 6). These results suggest that the interobserver reproducibility of CH planimetry may be greater than that of the GP method.

## Discussion

In our study, there were no significant differences noted in the areas between the right and left capitates and between the right and left hamates. Patterson et al. [15] reported that the volume of carpal bones was not significantly different between the left and right hands, using three-dimensional (3D) computed tomography (CT) volumetry. However, hand PA radiography for bone assessment is usually performed on the left side rather than on the right side, and perhaps because most people are right-handed, the right hand is more prone to get injured and therefore may have deformity. In the early 1900s, a physical anthropologists meeting determined that body measurements should be performed on the left [3].

The right capitate and bilateral hamates were not demonstrated on the radiograph in only one boy (0.41 %) aged 1 month. The capitate and hamate are known to emerge most frequently and simultaneously between the first and fourth months from birth; however, occasionally, the capitate emerges first, followed by the hamate. As seen in the GP atlas, the nuclei of the capitate and hamate were more likely to be present earlier in girls than in boys [16].

There was also a strong positive correlation between chronological age and the CH planimetry measurement (Table 3). As shown in Fig. 3, there were increased slopes in the planimetry curves of the capitates and hamates of boys and girls. Canovas et al. [17] reported that there was a strong correlation between chronological age and volumes of the carpal bones of the hands measured using 3D CT volumetry. The strongest correlations were found with the triquetrum, capitate and hamate bones, which were present in all 20 hands.

There was no significant difference between the two methods in accuracy for estimating bone age (CH planimetry, 84.39–84.46 %; GP method, 85.15–87.66 %). The research performed by Roche et al. showed that the median bone ages estimated using the GP method were similar to that of the chronological ages [18]. On the contrary, some researchers reported that the difference between chronological age and bone age estimated using the GP method was −0.6 to 2.15 years [10, 19–21]. The accuracy of BAA with the GP method has been reported to vary depending on the patient’s age or sex and ethnicity [22]. To date, few studies have evaluated the percentage accuracy of BAA using the GP method. Lee et al. [23] reported that a deep learning system for BAA achieved 57.32 % and 61.40 % accuracies for the female and male cohorts when age was precisely matched. The accuracy of matching bone age within 1 year was 90.39 % and 90.39 %, respectively.

In our study, the mean bone age measured using the GP method (105.49–109.49 months) was significantly lower than the mean bone age measured using CH planimetry (117.43–118.58 months). This result may be attributed to the difference in growth patterns among different ethnicities because the GP method was established in Caucasians [3]. Some researchers reported that bone age measured with the GP method tended to be lower than the chronological age or the bone age measured with the TW3 method or the Korean bone standard method, although there was no significant difference between the estimated bone age and chronological age [21, 24]. These previous studies may have shown no statistically significant difference because of the small number of children or targeting children of a limited age range.

In the second part of our study, the interobserver reproducibility of CH planimetry was greater than that of the GP method. The values of ρ_c_ were substantial for both methods (CH planimetry, 0.9862; GP method, 0.9553). In Bland-Altman analysis, the 95 % limit of agreement of CH planimetry was −10.5 to 13.4 months and that of the GP method was −21.1 to 29.5 months (Fig. 6). The precision of CH planimetry (4.42 % ± 3.87 %) was significantly different from that of the GP method (8.45 % ± 8.52 %) (*p* < 0.001). To date, there have been many comparative studies on the interobserver reproducibility of BAA with the GP method by using various statistical tests [5, 7]. Bull et al. [7] reported that the 95 % CI of the GP method (−2.46 to 2.18 years) was greater than that of the TW2 method (−1.48 to 1.43 years). King et al. [5] reported that the average spread of intra-observer variation was 0.96 years for the GP method. These results are similar to those of our study. By contrast, the percentage precision of the GP method was 65.5−88.5 % [8, 25]. Improving the observer variability of manual methods is a motivation for developing automated methods of BAA [26]. To date, computer-assisted or artificial intelligence-based methods have attempted to make accurate measurements, with discrepancies from 0.39 to 2.41 years [27].

This study has several limitations. First, it was performed retrospectively at a single institution. We excluded children with chronic illnesses, nutrition deficiency or growth problems from this study population. However, to our knowledge, this planimetric study is the largest series assessing the areas of the capitate and hamate.

Second, CT scans provide 3D measurements, whereas radiography demonstrates the 2D projected images of 3D objects; therefore, volumetry can be more accurate than planimetry, and may determine bone age more objectively [17]. However, CT volumetry has some disadvantages such as high cost and radiation exposure. In contrast, x-ray planimetry has the advantages of lower costs and lower radiation doses [28]. Moreover, planimetry is an easy-to-use method with an area measurement tool integrated in PACS without any separate software [29, 30].

Third, manually drawing ROIs along the cortical margin of the capitate and hamate may cause interobserver variation, and problems in planimetric analysis. To minimize this variation, another series of consensus measurements was performed in cases in which the differences between the reviewers exceeded 10 % [7, 31].

Fourth, quantitative analyses undertaken using digital radiographs displayed on a PACS monitor could be subject to measurement errors [32]. To avoid this error, to ensure the accuracy of the absolute measurement value, calibration was performed using a coin prior to our study [33]. Different equipment and protocols among different institutions may cause measurement errors. In the future, a multicentre prospective study should be conducted while avoiding this error.

Finally, there is no gold standard for BAA [29]. We set a reference standard with a range < 1 standard deviation of the Korea CDC growth chart. Children were enrolled in the reference standard only if their heights were between the 50th percentile of the pervious age stage and the 50th percentile of the later age stage, so that the height ranges do not overlap [12]. Moreover, as the height of Korean children has changed since the growth chart was published in 2007, it may be necessary to adjust the age range of the reference heights [11].

In conclusion, the CH planimetry method may be useful for BAA. In the future, if automated CH planimetry can be integrated in PACS with validation for ethnicity and sex, it may save time and allow more precise BAA.

## Abbreviations

CH: Capitohamate
GP: Greulich-Pyle
TW: Tanner-White
GR: Gilsanz-Ratibin
PACS: Picture Archiving and Communication System
ROI: Region of interest
